# An Interaction-Based Bayesian Network Framework for Surgical Workflow Segmentation

**DOI:** 10.3390/ijerph18126401

**Published:** 2021-06-13

**Authors:** Nana Luo, Atsushi Nara, Kiyoshi Izumi

**Affiliations:** 1 School of Geomatics and Urban Spatial Informatics, Beijing University of Civil Engineering and Architecture, Beijing 102612, China; lnn0331lnn@163.com; 2 Department of Geography, San Diego State University, San Diego, CA 92182-4493, USA; 3 Center for Human Dynamics in the Mobile Age, San Diego State University, San Diego, CA 92182-4493, USA; 4 Graduate School of Engineering, The University of Tokyo, Bunkyo-ku, Tokyo 113-8656, Japan; izumi@sys.t.u-tokyo.ac.jp

**Keywords:** surgical phases prediction, individual interaction measurement, bayesian network, zone position system

## Abstract

Recognizing and segmenting surgical workflow is important for assessing surgical skills as well as hospital effectiveness, and plays a crucial role in maintaining and improving surgical and healthcare systems. Most evidence supporting this remains signal-, video-, and/or image-based. Furthermore, casual evidence of the interaction between surgical staff remains challenging to gather and is largely absent. Here, we collected the real-time movement data of the surgical staff during a neurosurgery to explore cooperation networks among different surgical roles, namely surgeon, assistant nurse, scrub nurse, and anesthetist, and to segment surgical workflows to further assess surgical effectiveness. We installed a zone position system (ZPS) in an operating room (OR) to effectively record high-frequency high-resolution movements of all surgical staff. Measuring individual interactions in a closed, small area is difficult, and surgical workflow classification has uncertainties associated with the surgical staff in terms of their varied training and operation skills, patients in terms of their initial states and biological differences, and surgical procedures in terms of their complexities. We proposed an interaction-based framework to recognize the surgical workflow and integrated a Bayesian network (BN) to solve the uncertainty issues. Our results suggest that the proposed BN method demonstrates good performance with a high accuracy of 70%. Furthermore, it semantically explains the interaction and cooperation among surgical staff.

## 1. Introduction

In recent years, the evaluation of hospital effectiveness has gained increased attention with the introduction of new surgical techniques and procedures, such as surgical video database development and real-time tool-usage signal management [1,2,3,4,5,6]. In particular, there are studies aiming at recognizing and segmenting surgical workflow to assess efficiency inside an operating room (OR) and the skills of the surgical staff [7,8,9,10,11]. Furthermore, maintenance and improvement of surgical systems can benefit from surgical workflow monitoring and analysis, e.g., reducing surgical errors and better allocating health resources [12,13].

Various advanced approaches have been proposed to represent surgeries in an OR. These include collecting signals, videos, and images from a surgical procedure in an automatic way and using image preprocessing, speech recognition, and machine learning techniques to analyze and model this information [1,2,4,7,14,15,16]. James et al. developed a novel eye-gaze tracking technique to monitor eye movements underlying the cognitive processes of surgeons and their interactions with their surroundings [17]. To analyze surgical movement and gesture, Blum et al. detected 17 signals of tool usage in a laparoscopic cholecystectomy. The phases of the surgery were segmented to predict the remaining time of the surgical procedure [18]. Ahmadi et al. developed an automatic recovery of surgical workflow based on 17 signals from six different surgeries [19]. Leong et al. evaluated the representation of 3D trajectories of surgical instruments with hidden Markov models (HMMs) [20]. One key challenge in segmenting surgical procedures is data reduction. A large volume of information is often collected during a surgery with discriminative visual and spatial-temporal features. In the work of Giannarou and Yang, minimally invasive surgery videos were collected to assess surgical workflow. However, such valuable video data associated with high temporal redundancy requires a long time for visualization, making the analysis difficult [21]. To analyze and understand the surgical workflow with minimal information loss, they proposed a novel framework for a surgical representation to convey the content of the videos. The visual content information was generated by tracking 100 affine-invariant anisotropic regions. To solve the “extremely voluminous data” issue, Blum et al. extracted a variety of simple image features from laparoscopic video records to annotate which instrument was used at which time [18]. Then, the information regarding instrument usage was suggested as a dimension reduction in the raw video images, and further used to recognize the surgical phases.

Another challenge is that hospital systems have evolved into highly complicated, advanced, and technologically rich environments that increase the sophistication and complexity of surgical workflows, which leads to uncertainties in segmentation and recognition of the surgical phases [22]. For example, correct surgical performance and skills do not often produce satisfactory outcomes, partly due to the different initial status and biological conditions of the patients, even with the same degree of health improvements [5]. Even though surgical procedures are followed correctly, and regular steps are standardized, surgeons and other surgical staff never perform in the same way as their training and experiences vary individually [18]. Surgical procedures and the OR are complex systems with uncertainties because many individuals and units contribute to the surgical outcomes and health care system [5].

Despite the increasing interest among researchers in signal-, video-, and/or image-based surgical workflow segmentation, owing to high accuracy, casual evidence of individual interactions remains limited. Contrarily, the purpose of our study is to model surgical phases from individual interactions of surgical staff, and such interaction-based segmentation requires less voluminous data. To identify individual interactions of the surgical staff during a surgery, we installed a zone position system (ZPS) in an OR and collected high-frequency high-resolution movement data from all surgical staff. Such movement data highlight not only their movement trajectories and activity space, but also their interaction and cooperation [23,24]. This location-based staff interaction, including close-distance verbal communication, surgical instrument transfer, and collaborative surgical tasks, is unique to surgical phases, and can be used to characterize surgical workflows [25]. To cope with the uncertainty issues mentioned above, we integrated a Bayesian network (BN) that uses probabilistic reasoning to build a causal relationship between interactions of the surgical staff and surgical phases. We conducted the case study at the Tokyo Women’s Medical University (TWMU) in Japan, where we collected staff movement data from 10 neurosurgical operations, and developed a BN to segment the operations into six phases: ”Preparation”, ”Craniotomy”, ”Close”, “Magnetic Resonance Imaging” (MRI), “Tumor Resection” (TR), and ”End”. The results demonstrated that this novel interaction-based framework not only considers uncertainties regarding surgical procedure, surgical environment, surgery staff, and patients, but also provides a comprehensive, semantic explanation of the interaction and cooperation among the surgical staff.

## 2. Materials and Methods

### 2.1. Experiment Setup and Data Collection

As a case study, a neurosurgical operation in an OR at the TWMU was chosen to collect the real-time location of all surgical staff during the surgery with their written informed consent for participation. The OR room, where the ZPS is installed, had a dimension of 5.8 × 4.8 × 2.9 m The ZPS is an ultrasonic-based 3D location-aware system that is built on a surgical management system (SMS) offering real-time support for observing and recording surgical processes by collecting staff movement data. The ZPS consists of ultrasonic tags, receivers, and four control units. The ultrasonic tags are hooked at the back of each member of surgical staff and transmit signals, which are later detected by the receivers on the ceiling. Then, the four control units installed on the wall near the OR entrance detect the identification of each tag, as well as the location. At the TWMU, a neurosurgery is generally performed by four types of surgical staff: anesthetists, assistant nurses, scrub nurses, and surgeons. The accuracy of the ZPS for location tracking is 80 mm, and the sampling frequency is 50 Hz per tag. Ten cases of neurosurgical operations in 2007–2008 were chosen to model the surgical phases.

### 2.2. Interaction Measurement between Surgical Staff

In ORs, individual interactions play an essential role in recognizing surgical workflows and evaluating hospital efficiency [26,27,28,29,30]. As Flood et al. proposed, the characteristics of the corporate structure, where individual surgeons and other surgical staff organize themselves, attribute to the quality of surgical skills and workflow [5]. This study is conducted in an OR at the TWMU, with dimensions 5.8 × 4.8 × 2.9 m. Such a small, closed area presents the following issues: identifying individual interaction becomes difficult, and “fake” co-occurrences would be more prominent [31]. Here, a trajectory-based interaction simulation was conducted [32]. We calculated the trajectory similarities between any two surgical staff using the longest common subsequence (LCSS) and interpreted the computed similarity values as the interaction and cooperation of two surgical staff.

### 2.3. Bayesian Network-Based Surgical Phase Classification

With the increased sophistication of surgical instruments and techniques, surgeries and ORs have become a complex system where the differences among surgical staff (e.g., training and operation skills) and variance among patients (e.g., initial health status, age, and gender) lead to uncertainty and complexity in the recognition and segmentation of surgical workflow. The Bayesian network, a probabilistic reasoning methodology, can be applied to solve such uncertainty and complexity. BNs are graphical models that reason and infer under uncertainty, where the nodes represent the variables of interest, both continuous and discrete, and the directed arcs between any pair of nodes represent the strength of the connection between them. BNs use probabilistic beliefs to qualify the connections and update the strength automatically based on the input of new evidence.

BNs are commonly understood as a representation of the joint probability distribution of the variables/nodes involved [33]. Consider a BN with n nodes $X_{1},{\;X}_{2},\ldots ,{\;X}_{n}$. The joint distribution is computed by $P(X_{1},{\;X}_{2},\ldots ,{\;X}_{n}$) at a particular value, e.g., at $X_{1}=x_{1},{\;X}_{2}=x_{2},\ldots ,{\;X}_{n}=x_{n}$. Based on the chain rule of probability theory, the joint probability $P(X_{1},{\;X}_{2},\ldots ,{\;X}_{n}$) is factorized as follows [34]:

(1) $$\begin{array}{cc} P(X_{1},{\;X}_{2},\ldots ,{\;X}_{n}) & =P(X_{1}=x_{1})\ldots \times \;P(X_{n}=x_{n}|X_{n-1}={\;x}_{n-1},\ldots ,X_{1}=x_{1})\\ & =\underset{i}{\prod }p(X_{i}|\mathrm{Parents}\left(X_{i}\right)) \end{array}$$

The basic task of a BN is to compute the posterior distribution of a query node, given the evidence input of other nodes. Then, the value for the query node is estimated. Consider two nodes as an example, X->Y:

(2) $$\mathrm{Posterior}\left(X\right)=P\left(X|Y\right)=\frac{P\left(X\right)\times P(Y|X)}{P\left(Y\right)}$$

where P(X) is the prior distribution of a query node and P(Y|X) is the likelihood of the query node.

Given these data, the structure of a BN is learned first. Many methods have been developed to learn the structure, and the learning methods are generally classified into two types: score-based structural learning and constraint-based structural learning [35,36,37]. The constraint-based learning method, which identifies the conditional independence between nodes, is popular and close to the semantics of the BN. Furthermore, most structure learning algorithms belong to the score-search type, where a search algorithm is created to seek through the space of all possible BNs, and a score function that considers not only the model fit to data, but also the complexity of the model is defined to measure the quality of the candidate BN [38]. Consider a BN with a graph G and dataset D:

(3) $$\mathrm{score}\left(G,D\right)=P\left(G|D\right)=\frac{P\left(D|G\right)\times P\left(G\right)}{P\left(D\right)}$$

where P(D) does not depend on G and P(G) is the prior information [28]. Because the score-based method attempts to maximize the score function, P(G) can be ignored, and the key parameter is P(D|G). Different score functions have various equations for P(D|G). Here, a maximum likelihood estimation was used to define the score function.

Although learning a BN structure is known to be computationally intense, the hill climbing algorithm (HC) is particularly efficient and popular because of its good trade-off between model fit with respect to data and computational demand [39,40]. The HC traverses the space of all candidates by starting from an initial network and performs a local change at each step, such as adding, deleting, and reversing the arc. Given a well-defined score function, the score of the new BN is computed, and finally, the BN with the highest score is identified. Once the optimal structure is known, the parameters are learned to estimate their posterior distribution, given the new evidence predicting the possible value of the query variable. We use the maximum likelihood estimation:

(4) $$L\left(\theta |D\right)=P\left(D|\theta \right)=\prod_{i}P(X_{i}=x_{i}|\mathrm{Parents}\left(X_{i}\right)=a,\theta )$$

where $x_{i}$ is the state for the node $X_{i}$ and a is the state for combination of the node $X_{i}$’s parent nodes.

BNs, generally known as casual models, explanatory models, or predictive models, have been applied to a variety of problems such as casual reasoning, regression, and prediction, as well classification problems [41]. In other words, a BN classifier is learned using the training data consisting of a target node T and a set of attribute nodes $A_{i}$, and it is used to classify the target node T based on new evidence and the learned BN.

(5) $$\begin{array}{cc} P\left({T|A}_{1},\ldots ,A_{n}\right) & =\frac{P\left(T,A_{1},\ldots ,A_{n}\right)}{P\left(A_{1},\ldots ,A_{n}\right)}=\frac{\mathrm{BN}}{P\left(A_{1},\ldots ,A_{n}\right)}\\ & =\frac{\prod_{i}p(X_{i}|\mathrm{Parents}\left(X_{i}\right))}{P\left(A_{1},\ldots ,A_{n}\right)} \end{array}$$

where $X_{i}\in U\left(T\cup A_{i}\right)$ [42].

## 3. Results

### 3.1. Spatial and Temporal Patterns between Different Surgical Staff

Here, the ZPS was installed to observe and record the surgical processes in a real-time manner and collect the associated surgical staff movement data with high frequency and high resolution. In general, such space-time movement presents two types of information: the activity space of an individual and time. Visualizing individual movements in space-time can reveal peoples’ spatial and temporal availability [31,32,43,44,45].

**Figure 1:**
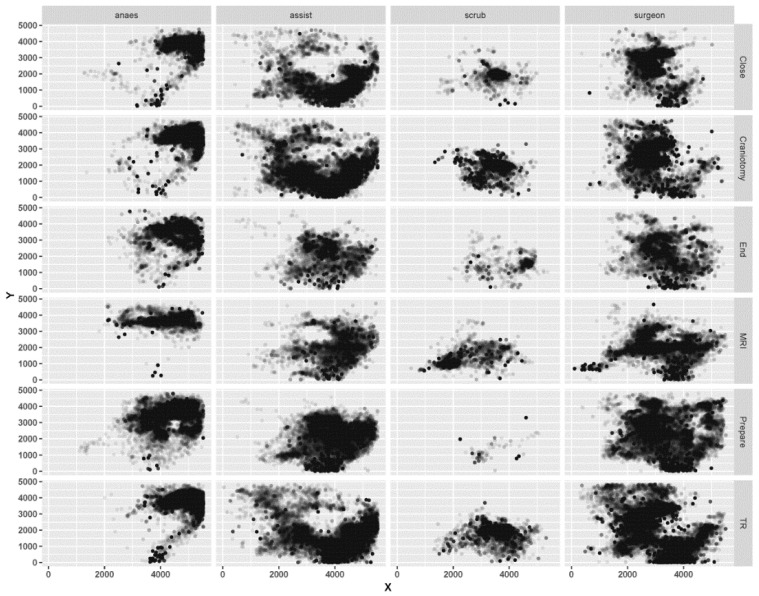
Spatial patterns of the surgical staff during surgery.

Figure 1 depicts the movement patterns of the surgical staff, which is a two-dimensional space, namely, location (X, Y). We can clearly see the spatial patterns and activity space for all surgical roles during surgery. Significant differences in the activity space were found between the surgical roles and phases. Surgeons and assistant nurses have the largest activity space, both located in the center of the OR. The surgeons move nearly throughout the entire room, while the assistant nurses move mainly around the bottom of the room. The activity space for the anesthetists is at the upper-right corner of the OR. The scrub nurses have the smallest moving space, at the center of the room, and they seldom appear in the OR during the “Preparation”. In the surgical phase, a specific movement pattern is identified. The entrance to the OR lies at the bottom of the room, at (4000, 0), and the surgical tool is located at the upper left corner of the room, at (1000, 3000). During the “MRI”, surgeons and anesthetists have notable horizontal movements. During the “TR”, “Craniotomy”, and “Close”, surgeons undertake multiple sophisticated surgical operations, which require help from assistant nurses, in tasks such as transferring surgical tools. Therefore, during these phases, assistant nurses demonstrate considerable movement around the tool area.

**Figure 2:**
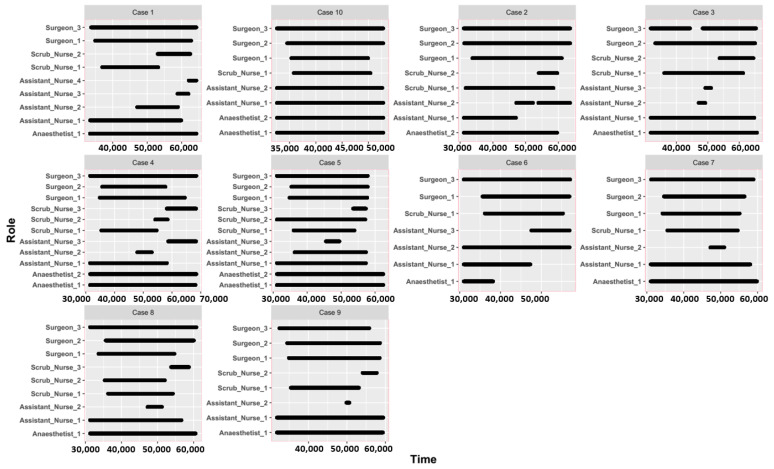
Temporal patterns of the surgical staff during surgery.

Figure 2 describes the temporal scale and time flexibility of the surgical staff during surgery, for example, when an individual staff member works or how flexible their time is. There are notable differences among surgical staff. Although each surgery has a varied number of surgical staff, the surgeons, assistant nurses, and anesthetists generally work throughout the surgery timespan, while scrub nurses have greater flexibility at the beginning and end of the surgery. Evidently, such variances increase the uncertainty in recognizing and predicting the surgical workflow.

**Figure 3:**
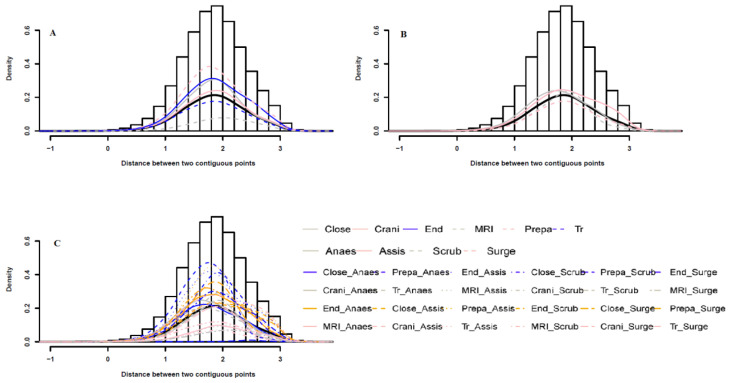
Probability distributions of distance between any two continuous points per surgical role per surgical phase. (**A**) probability distributions of distance per surgical phase; (**B**) probability distributions of distance per surgical role; (**C**) probability distributions of distance per surgical role per surgical phase.

The probability distribution of the distance between two contiguous points per surgical role per surgical phase is shown in Figure 3. The peak represents the mean value. The ZPS sampling is performed every second, therefore, the distance between two consecutive points is interpreted as speed. In the phase-oriented probability distribution A, the maximum probability is the largest for the “Preparation” and the lowest for the “MRI”, which suggests that during “Preparation” the staff moves slower than during “MRI”. In the role-based probability B, the distributions per role exhibit a similar trend. In the phase- and role- based probability C, the anesthetist movement is the slowest during “Preparation”, while the assistant nurse movement during “Craniotomy” is the fastest.

### 3.2. Trajectories and Interactions between Different Surgical Staff

**Figure 4:**
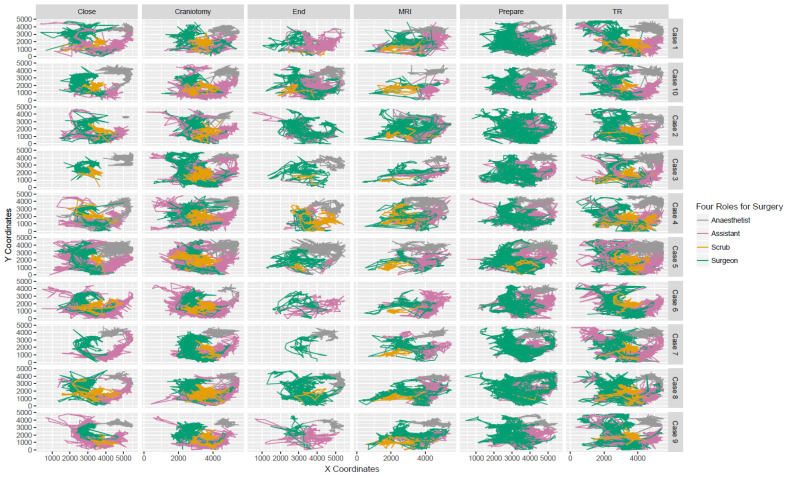
Movement Trajectories per surgical role per surgical phase.

Figure 4 shows the movement trajectories of the surgical staff during surgery. It is difficult to measure individual interactions in a small area [28,29,30]. Here, the selected OR is a small, closed area, which raises a significant challenge in using point-based geographical co-occurrence to measure individual interactions: “fake” co-occurrence is expected [25]. As shown in Figure 4, the surgical staff, including the surgeons (green line), assistant nurses (pink line), scrub nurses (orange line), and anesthetists (gray line), have varying trajectories per case. During “Close”, the trajectories of surgeons in cases 3 and 7 are completely different from other cases. Nevertheless, the trajectories of surgeons and scrub nurses still overlap when they interact with each other. During “End”, the trajectories of anesthetists vary largely, almost missing in cases 2 and 6, but overlap with the other staff. Here, such a trajectory overlap is interpreted as the interaction between the surgical staff. In Figure 4, the trajectories consist of both the location information and corresponding timestamp, and the similarity between the trajectories of any two staff is computed to represent the interaction among them.

**Figure 5:**
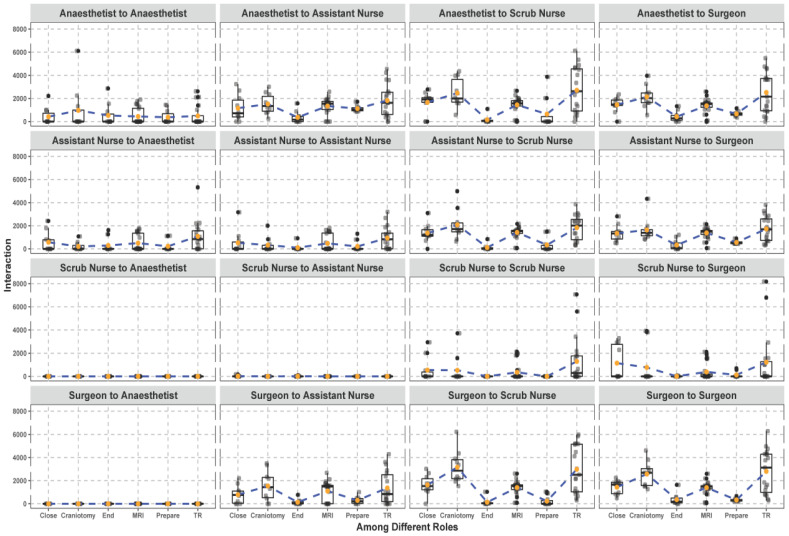
Trajectory-based interaction measurement between the surgical roles during surgery.

The results of the trajectory similarity computations are shown in Figure 5. Sixteen kinds of interaction were measured, and we clearly identified who interacts with whom and how the interaction varied per surgical phase. We also detected the interaction network for each surgical role. The scrub nurses had a smaller interaction network than the anesthetists and assistant nurses, while the surgeons ranked third. The scrub nurses did not interact with the anesthetists and assistant nurses, and the surgeons had no connection to the anesthetists. Furthermore, the interaction between the surgeons and scrub nurses had a larger variation compared to that of the assistant nurses, which is probably due to the intermittent participation of intern surgeons in surgeries. Finally, the interactions between the anesthetists and scrub nurses, the anesthetists and surgeons, and the surgeons and scrub nurses are similar during each surgical phase.

### 3.3. Bayesian Network-Based Surgical Phase Classification

**Figure 6:**
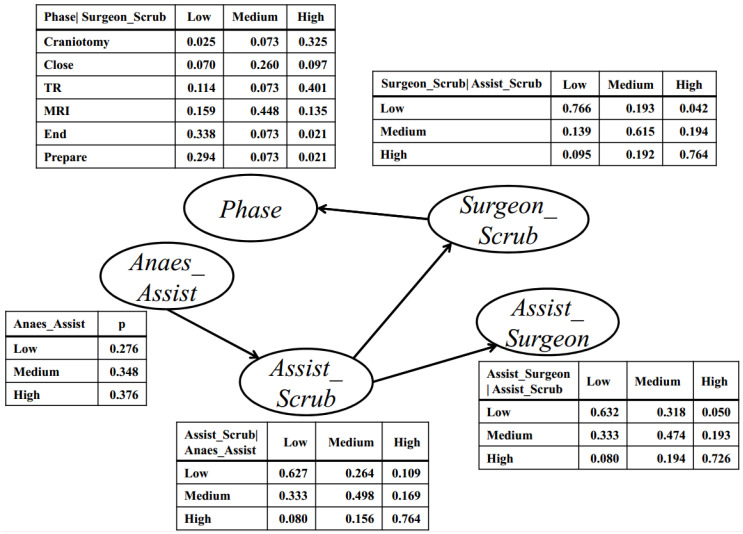
The learned BN structure and conditional probability table.

To segment the surgical phases using a BN, the interactions between the surgeons and scrub nurses, the assistant nurses and surgeons, the anesthetists and assistant nurses, and the assistant nurses and scrub nurses were selected to learn the structure. Figure 6 depicts the learned structure and conditional probability tables (CPTs). Each node represents one kind of interaction that has three states discretized from the continuous LCSS results: “Low”, “Medium”, and “High”. The directed links represent the casual relationships between the nodes, and the CPT represents the probability that one node is in a specific state given the states of its parent nodes. In the CPT, the conditional probability for the “phase” node, given the interaction between the surgeons and scrub nurses, is distributed by six phases and three interaction levels. These conditional probabilities that are stored in a CPT show the strength of the relationships between two connected nodes.

As shown in Figure 6, the interaction between the surgeons and scrub nurses directly determines the surgical phases. The “Craniotomy” and “TR” are related to frequent interactions of the surgeons and scrub nurses, while the “End” and “Prepare” are related to less frequent interactions of the surgeons and scrub nurses. This result suggests that the “Craniotomy” and “TR” experience high cooperation between the surgeons and scrub nurses, whereas an opposite trend is observed in the “End” and “Prepare”. The interaction between the surgeons and scrub nurses is also influenced by the interaction between the assistant nurses and scrub nurses. When the surgeons and scrub nurses interact, the assistant nurses and scrub nurses interact as well. During “Craniotomy” and “TR”, the surgeons and scrub nurses interact, which still needs contribution from the assistant nurses, such as transferring surgical tools. In these two phases, the surgeons, scrub nurses, and assistant nurses work closely. Although the interactions between the assistant nurses and surgeons, and the anesthetists and assistant nurses, have little direct influence on the surgical phases, they affect the interaction between the surgeons and scrub nurses, and indirectly influence the surgery. When the scrub nurses interact with the assistant nurses, it is generally during the “Craniotomy” or “TR” phase where the anesthetists work on the patients. The interactions between the surgical roles and their relationship with surgical phases can be explained semantically by the BN.

The CPT describes the prior probability distribution of each interaction and surgical phase and computes the posterior probability distribution of each phase using the new evidence input, that is, the surgical phase classification, also called “probability updating” The phase is predicted based on the parent variable and the interaction between the surgeons and scrub nurses, followed by the calculation of its posterior probability.

**Figure 7:**
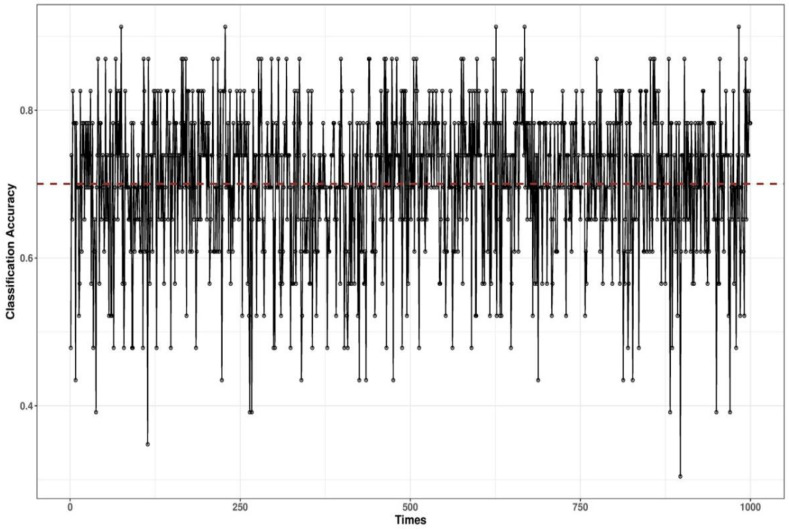
Validation for the BN-based surgical phase classification.

**Figure 8:**
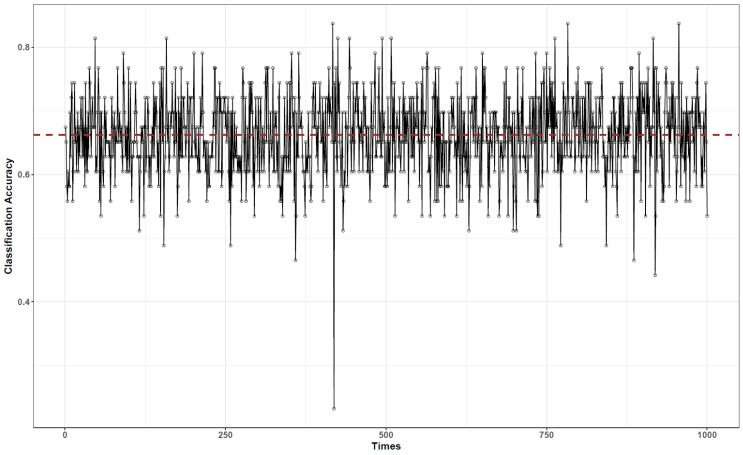
Validation for the Naive Bayes-based surgical phase classification.

To validate the classification results, we conducted cross-validation. The data were randomly divided into training and validation datasets: 40 of the 83 data points were used for training and the remaining 43 for validation. This validation was run 1000 times to reduce the impact of the data assignment on training and validation. The results are shown in Figure 7. The accuracy varies from 0.4 to 0.85, and the average accuracy is 0.7. To validate the BN, we also performed a naive Bayes-based classification, and the result is shown in Figure 8. As observed, the naive Bayes-based classification has a similar accuracy of 0.68, but the naive structure cannot semantically explain the interaction between the surgical roles and their relationship with surgical phases.

## 4. Discussion

Here, we presented a reliable way to recognize the phases of a neurosurgical operation using only surgical staff movement data. A series of movement features, such as individual interactions, spatial and temporal patterns, movement trajectories, and probability distributions of the distance between two consecutive points, are able to be extracted. We found that these features are unique to each surgical phase, and therefore, are able to characterize the surgical workflow as well as evaluate the surgical effectiveness. Movement trajectories can also reveal the activity space of an individual. Visualizing the movement trajectories of the surgical staff helps identifying the “available and empty” area and time in ORs. It further improves the space utilization of the ORs, such as relocation of surgical instruments and rearrangement of surgical staff. In the case study, the corners and left area of the OR were found to be available during the surgery and relocating surgical instruments and intern students in these areas helped improve the effectiveness of the surgery. We also found that the interaction between the surgical staff has a large variance across the surgical phases, which usually fails to be captured by visual and signal data. The identification of this variance has a growing importance in evaluating the efficiency of surgical procedures and resource usage.

The traditional method to measure individual interaction is based on geographical co-occurrence: two individuals being in the same location at the same time, approximately. However, the movement data used in this study were collected in an OR, which increases the complexity and inaccuracy in determining the spatial and temporal thresholds to identify the co-occurrence. A novel representation of individual interactions was proposed, namely the trajectory-based interaction measurement. Here, the interaction between two individuals is computed based on their movement trajectories, and the trajectory similarity suggests the extent of their interaction and cooperation. We calculated 16 types of interactions between the surgical staff and identified the interactions specific to each surgical phase. The “Craniotomy” and “TR” are closely related to frequent interactions of the surgeons and scrub nurses, while the “End” and “Prepare” are related to less frequent interactions of the surgeons and scrub nurses. Compared to the “Craniotomy” and “TR”, the “End” and “Prepare” experience less interaction and cooperation, which creates the potential to reallocate labor resource, e.g., adjusting the minimum number of surgical staff to perform a surgical task effectively.

Currently, surgical phase classification remains challenging and uncertain with regard to the complex nature of ORs and surgical processes, initial states and biological differences of patients, differences in surgical procedures, and varied experience and surgical skills of surgical staff. Nevertheless, we proposed a BN-based surgical phase classification that incorporated such uncertainty by inferring the surgical phases from probability reasoning. To improve the performance of the learned BNs and the accuracy of the surgical phase classification, additional movement features, such as movement trajectories and activity space, must be included into the BNs, which will ultimately lead to the development of interaction-based surgical phase classification with high dependability. In the validation, compared to naive Bayes, our BN with an average accuracy of 70% shows a better classification performance. Most importantly, our BN provides a semantic explanation and understanding of the interactions among surgical staff, as well as their relationship with the surgical phases.

## 5. Conclusions

Segmenting and representing surgical workflow not only helps to evaluate hospital and surgical efficiency, but also improves the surgical processes and resource usage. Two types of data, visual and signal data, are typically used to perform this task. Inimitably, here, we collected real-time movement data of the surgical staff in the TWMU ORs and explored the causal relationships between the interactions of the surgical staff and neurosurgeries. We found that such interactions can explain 70% of the surgeries and proposed that surgical staff movement data in addition to signals, videos, and/or images of surgeries can be used segment and understand the surgical workflow.

## Data Availability

Not applicable.
